# 

**Published:** 1889-07

**Authors:** 


### SOCIETY NOTES.

### MISSISSIPPI VALLEY MEDICAL ASSOCIATION.

The next annual meeting of this Association will be held at Evansville, Ind., September 11th, 12th and 13th, 1889. The officers for this year are:

President, Dr. George J. Cook, Indianapolis, Indiana.

Vice Presidents, Dr. J. D. Griffiths, Kansas City, Mo., and Dr. J. A. Larrabee, Louisville, Ky.

Secretary, Dr. R. L. Thomson, St. Louis, Mo.

Treasurer, Dr. W. C. Chapman, Toledo, Ohio.

Chairman of Committee of Arrangements, Dr. A. M. Owen, Evansville, Ind.

This Association, the offspring of the Tri-State Society (Indiana, Kentucky and Illinois,) was organized under the present name in 1883, and it is rapidly attaining the object of its formation—a thorough organization of the members of the regular medical profession of the entire Mississippi valley; thus to foster, advance and disseminate medical knowledge, and to uphold the honor and maintain the dignity of the medical profession.

Every member of the regular profession in good standing can become a member of this Association, and all are earnestly invited to attend this year.

The importance of an association to bring together the medical profession within this territory must be apparent to every one, as there are many interests in common, and individual welfare can best be promoted by the advancement of the interests of all.

Evansville was selected as a place of meeting on account of its central location, being easily accessible from all points, by river and by rail.

Questions of importance to the entire profession will be before the Association for consideration at the coming meeting, and it is especially for the officers to know that the indications are that the coming meeting will be larger than any yet held by the Association.

Already the committee of arrangements are actively at work arranging for a royal reception of the society in September.

Many papers have been promised by prominent members of the profession. Those who desire to place before the Association the results of their observation and experience in practice and scientific investigation of medical subjects should send the titles of papers to the chairman of the committee of arrangements, or to the secretary at as early a date as practicable.

